# Digital Twins for Managing Health Care Systems: Rapid Literature Review

**DOI:** 10.2196/37641

**Published:** 2022-08-16

**Authors:** Safa Elkefi, Onur Asan

**Affiliations:** 1 School of Systems and Enterprises Stevens Institute of Technology Hoboken, NJ United States

**Keywords:** health care, digital twins, safety, information management, supply chain management, operational control, well-being promotion, human factors, technology, health informatics, literature synthesis, scheduling and optimization, digital health

## Abstract

**Background:**

Although most digital twin (DT) applications for health care have emerged in precision medicine, DTs can potentially support the overall health care process. DTs (twinned systems, processes, and products) can be used to optimize flows, improve performance, improve health outcomes, and improve the experiences of patients, doctors, and other stakeholders with minimal risk.

**Objective:**

This paper aims to review applications of DT systems, products, and processes as well as analyze the potential of these applications for improving health care management and the challenges associated with this emerging technology.

**Methods:**

We performed a rapid review of the literature and reported available studies on DTs and their applications in health care management. We searched 5 databases for studies published between January 2002 and January 2022 and included peer-reviewed studies written in English. We excluded studies reporting DT usage to support health care practice (organ transplant, precision medicine, etc). Studies were analyzed based on their contribution toward DT technology to improve user experience in health care from human factors and systems engineering perspectives, accounting for the type of impact (product, process, or performance/system level). Challenges related to the adoption of DTs were also summarized.

**Results:**

The DT-related studies aimed at managing health care systems have been growing over time from 0 studies in 2002 to 17 in 2022, with 7 published in 2021 (N=17 studies). The findings reported on applications categorized by DT type (system: n=8; process: n=5; product: n=4) and their contributions or functions. We identified 4 main functions of DTs in health care management including safety management (n=3), information management (n=2), health management and well-being promotion (n=3), and operational control (n=9). DTs used in health care systems management have the potential to avoid unintended or unexpected harm to people during the provision of health care processes. They also can help identify crisis-related threats to a system and control the impacts. In addition, DTs ensure privacy, security, and real-time information access to all stakeholders. Furthermore, they are beneficial in empowering self-care abilities by enabling health management practices and providing high system efficiency levels by ensuring that health care facilities run smoothly and offer high-quality care to every patient.

**Conclusions:**

The use of DTs for health care systems management is an emerging topic. This can be seen in the limited literature supporting this technology. However, DTs are increasingly being used to ensure patient safety and well-being in an organized system. Thus, further studies aiming to address the challenges of health care systems challenges and improve their performance should investigate the potential of DT technology. In addition, such technologies should embed human factors and ergonomics principles to ensure better design and more successful impact on patient and doctor experiences.

## Introduction

One of the fastest growing sectors of the global economy is the health care industry [1,2]. For a complex system like a hospital, many problems and obstacles arise owing to the variability resulting from the incongruity between demand, and capacity and resource availability. In addition to the operational management of resources, having an almost instantaneous and reliable vision of the available resources would permit a more adaptive management of the resources as the demand varies. As the demand changes, managing staff schedules, patient flow, bed sizes, and room usage would be a challenge [1]. Technology-based strategies can be promising contributors to improving the efficiency of health care delivery. The increasing adoption of various health information technologies has created new channels for management [3] and communication [4] that revolutionize health care systems.

**Figure 1:**
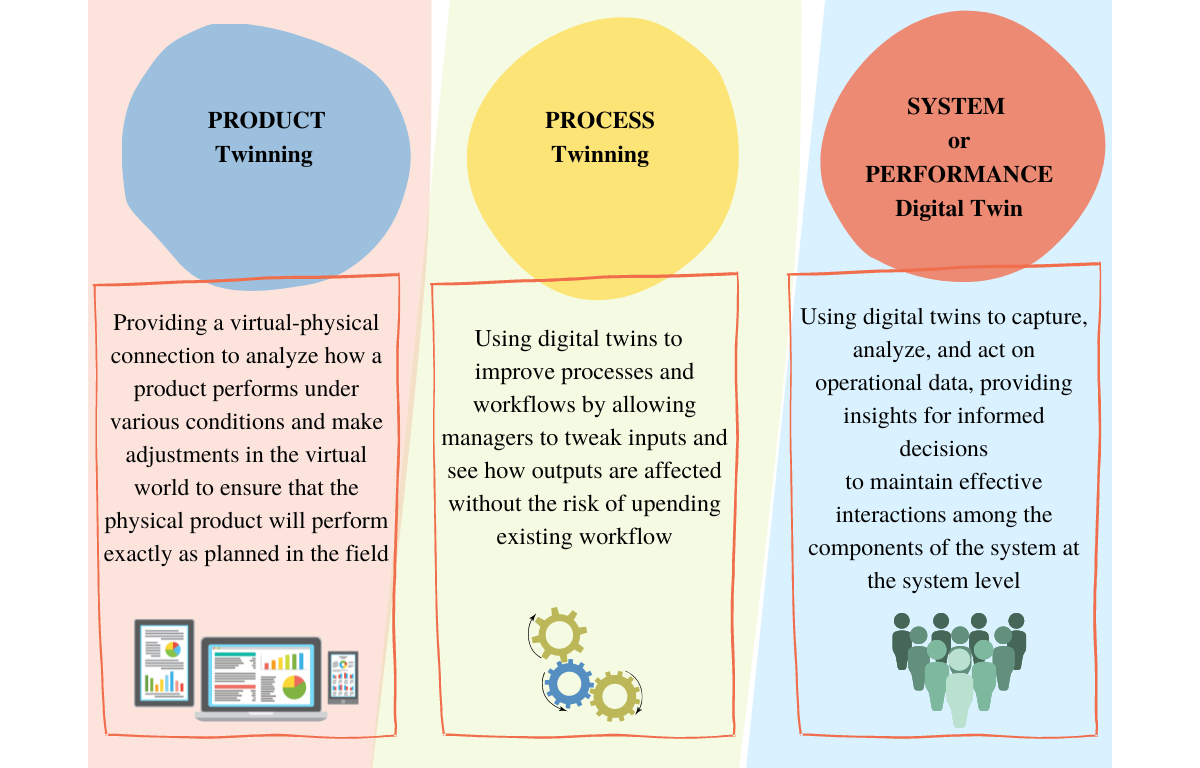
Types of digital twins.

Meanwhile, a revolution toward an intelligent industry or “Industry 4.0” combining advanced technologies emerged in 2011 [5]. This revolution affected all sectors, including health care. One of the supporting concepts in implementing Industry 4.0 is the digital twin (DT) [6]. A DT is a virtual representation of a physical asset replicated virtually through data connection [7,8], making it possible to link the system with its virtual copies in a bidirectional way [9]. Digital technologies provide many opportunities to revolutionize health care. For example, real-time data can be provided by Internet of Things solutions, and large data flows are managed and secured by robust digital infrastructures. In addition, flows and decision-making support are improved by command centers, artificial intelligence, and machine learning [10-12]. However, it is through the creation of DTs that much of this can be brought together [8]. The medical DT concept is considered more beneficial for personalized medicine to help health care professionals realize more effective interventions by digitally replicating the human body, allowing prevention, early detection, and targeted treatments of many diseases [13,14]. This paradigm is not limited to medical practice improvement; it also offers a solution to the issues related to health care systems and supports their strategic management. A DT can help design, optimize, and test products; design and operate production systems; manage and control supply chains; diagnose problems; and provide after-market services [15]. There are 3 types of DTs, as illustrated in Figure 1.

In this context, this rapid review aims to highlight what DTs have accomplished in correlation with health care management support. We intend to cover the interventions that used DTs (products, processes, systems) to improve the management of medical services. We report the DT type and its role in the system (function). This classification of DTs is adopted from Siemens, classifying DTs into 3 types, with 1 related to processes (eg, production), 1 related to product design, and 1 related to system performance (eg, performance) [16]. The combination and integration of the 3 DTs as they evolve together is known as the digital thread [16].

## Methods

### Study Design

We performed a rapid review of studies involving DT technology to improve health care services management. Rapid reviews are a form of evidence synthesis that can provide timelier decision-making information than standard systematic reviews. They are suitable for emerging research topics where systematic reviews are unpractical [17]. Rapid reviews typically do not include an exhaustive set of studies, do not involve formal analyses of the study quality, and report findings from prior studies via narrative synthesis by simplifying the evidence synthesis process [18].

Our protocol was registered on January 28, 2022, with the Open Science Framework [19]. The primary goal of this review was to identify the opportunities that DTs have offered to support the improvement of the health care system. We summarized the literature on existing applications of DTs and the challenges associated with their design, use, and implementation. Publications spanning the last 20 years were considered, from January 1, 2002, to January 25, 2022. We started in January 2002 because the concept of DTs was publicly first introduced in 2002 by Grieves [20]. Grieves proposed the DT as the conceptual model underlying product lifecycle management [20].

### Search Strategy

We searched PubMed, Web of Science, IEEE Xplore, Scopus, and ScienceDirect using “digital twin” and “health” as the keywords. The studies included journal and conference articles that covered only the health care applications of DTs (no industrial, manufacturing, or energy-related initiatives). We excluded the following types of studies: studies published in a language other than English; reviews, short communications, and briefs not reporting the impact of DTs through empirical studies and approaches; papers that are not peer-reviewed; studies that present an initiative to support a medical practice (precision medicine, organ transplant, etc).

Studies are discussed based on the contribution of DTs to improving user experience in health care from human factors and systems engineering perspectives, accounting for the type of impact (product, process, or performance/system level).

## Results

**Figure 2:**
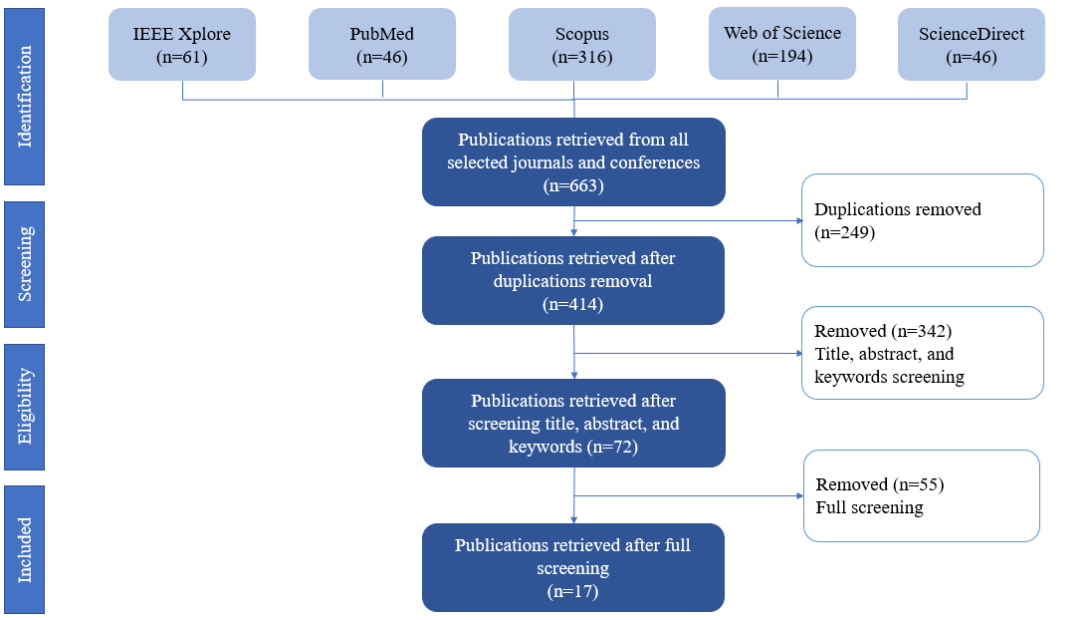
PRISMA (Preferred Reporting Items for Systematic reviews and Meta-Analyses) flow diagram of the article selection process.

Of all the sources found, 72 papers were screened comprehensively, and 17 papers were included in this review. The screening process is summarized in the PRISMA (Preferred Reporting Items for Systematic Reviews and Meta-Analyses) flow diagram shown in Figure 2.

Even though very few articles matched our scope, the research trend is evolving. The selected papers covered different application areas, including process development (n=5), a system improvement initiative (n=8), and developing/designing and testing a product (n=4). We adhered to conventions for narrative reviews by combining our results with interpretations and discussion in the Results and Discussion sections.

We identified 4 main functions that DTs perform in managing health care systems. We summarize the functions and their adopted definitions in Table 1.

**Table 1:** Functions of digital twins identified in this study.

Function	Definition
Safety management	Avoiding unintended or unexpected harm to people during the provision of health care processes; identifying crisis-related threats to a system and helping control the impacts
Operational control	Ensuring high system efficiency levels by making sure that health care facilities run smoothly and offer high-quality care to every patient
Information management	Ensuring privacy, security, and real-time information access to stakeholders
Health management and well-being promotion	Empowering self-care abilities by enabling health management practices

These technologies have the potential to avoid unintended or unexpected harm to people during the provision of health care processes and identify crisis-related threats to a system and help control the impacts, which we define as safety management. They are also used to ensure privacy, security, and real-time information access to all stakeholders, which can be considered information management. Another function identified is well-being promotion and health management, where DTs empower the self-care abilities of patients by enabling health management practices. The last function is operational control and management. This technology has the potential to ensure a high system efficiency level by ensuring that health care facilities run smoothly and offer high-quality care to every patient.

Table 2 summarizes the functions of DTs, along with the key findings of the studies considered in this review.

Our findings are presented per the function of DTs (safety management, information management, health management and well-being promotion, and operational control). The operational control findings are presented in 5 subparts (process control of anomalies, scheduling of interventions, resource allocation, operation optimization, and strategy optimization). After presenting the state of the art of DT use, we present the challenges associated with the use and implementation of DTs, followed by the limitations of our study.

**Table 2:** Functions and key findings.

Function and type of DT^a^			Key findings
**Safety management**			
	System (Jovanović et al, 2021) [21]	The DT used allows management of the infection peak and development of precisely targeted vaccination strategies that allow targeting based on individuals' number of social contacts.	
	System (Talukder, 2021) [22]	A DT architecture is proposed to ensure safety within an ecosystem disrupted by COVID-19. It mitigates the system challenges and increases patient safety in post–COVID-19 health care delivery.	
	System (Alrashed et al, 2022) [23]	A DT was used to simulate the different possible strategies and scenarios to predict the spread of the COVID-19 virus and minimize the impacts while ensuring continuity in providing services to citizens.	
**Information management**			
	Product (Lutze, 2019) [24]	DTs embedded in wearable devices were used to gather personal information to make group or system decisions.	
	System (Pang et al, 2021) [25]	A city DT was developed based on federated learning principles to serve as a local central server of information. It allows centralizing information, sharing knowledge, sharing local strategies, and sharing responses to adopted plans in real time among multiple cities.	
**Health management and well-being promotion**			
	Product (Díaz et al, 2021) [26]	The DT developed (DTCoach) serves as not only an accompanying educator but also as a mentor that can be used on portable devices like smartphones. It enables web-based pose estimation and performance measurement by providing a person-centered digital coaching experience with a platform that serves as a coach, an accompanying educator, and a mentor who can help make the necessary adjustments based on the individuals' capabilities.	
	Product (Liu et al, 2019) [27]	The suggested cloud-based system, ClouDTH, uses personal data from digitally twinned wearable medical devices to achieve interaction and convergence between physical and virtual medical spaces to facilitate personal health management for elderly patients.	
	Product (Tröbinger et al, 2021) [28]	The DT product developed serves as an alternative to telemedicine solutions. It presents a new approach to the remote doctor visit. The dual doctor-patient twin paradigm involves 2 robotic systems (patient GARMI, doctor MUCKI). Control, interaction, and knowledge transfer are enhanced by artificial intelligence, visual motion, and facial expression analysis in the DT. Thus, it enables a transparent remote doctor visit and better-informed and robot-assisted telerehabilitation with bidirectional telepresence control.	
**Operational control**			
	Process (Nonnemann et al, 2019) [29]	DTs of the processes in an ICU^b^ station are integrated into a system (Health@Hand) to allow remote monitoring; it detects faults and anomalies immediately and will enable interventions at an early stage.	
	Process (Chase et al, 2021) [30]	DTs of the processes are used to optimize the interventions in a medical ICU. They aim to optimize patient care by clinical staff at the enterprise level for more productivity and quality improvement.	
	System **(**Karakra et al, 2018) [31]	The hospital's DT proposed developing a predictive decision support model that employs real-time services data drawn from these systems and devices. This model enables assessing the efficiency of existing health care delivery systems and evaluating the impact of changes in services without disrupting the daily activities of the hospital. It allows foreseeing the effectiveness of changes in the models before they are applied in reality.	
	System (Augusto et al, 2018) [32]	The DT of an emergency unit is developed to optimize the pathway of patient care in the unit. The system accounts for various arrival processes to account for massive arrivals in case of a crisis and determine the best available leverages to optimize the operations of the system.	
	Process (Mylrea et al, 2021) [33]	BioSecure DT monitors every step in the supply chain process to ensure good productivity and cybersecurity by applying Cyber-Informed Engineering.	
	Process (Karakra et al, 2019) [34]	It predicts the near future and monitors the processes in real time through the HospiT'Win (DT) system. It allows detection of unexpected situations before problems occur in real life (delay, change in schedule, etc). It will enable the tracking of data flow from the real world to the virtual world.	
	System (Karakra et al, 2020) [35]	Using discrete event simulation and DTs through a system called HospiT'Win allows tracking the pathways of patients inside the health care organization to manage growing demand and decrease waiting times and delays. The solution enhances resilience to sustain critical operations under expected and unexpected conditions. It conveys key information to decision-makers in real time.	
	System (Rodríguez-Aguilar et al, 2020) [36]	The DT of the health care system is developed to better respond to contingencies and ensure optimal allocation of available resources in a DHPES^c^.	
	Process (Croatti et al, 2020) [37]	A trauma DT is used to digitalize and support the process of severe trauma management, considering it as a physical asset that is mirrored by 2 DTs.	

^a^DT: digital twin.

^b^ICU: intensive care unit.

^c^DHPES: Digital Health Public Emergency System.

## Discussion

### State of the Art of DT Usage

Health care has evolved away from focusing solely on illness toward primary health care and health promotion, considering health care as a complex ecosystem [38]. The most significant contributions of digital twinning in health care have been precision medicine efforts that provide patients with targeted treatment and diagnosis [39]. However, its use to develop novel customized health care management approaches started in 2018 and is still an evolving concept [40].

#### Safety Management

The goal of the patient safety movement is to reduce adverse outcomes or injuries resulting from health care processes. It is imperative that these adverse outcomes are avoided, prevented, or minimized [41]. With the improvements in safety standards and policies, more attention is accorded to analyzing safety issues and the sources of these issues [42]. Errors and inefficiencies in inpatient care are frequently the results of conflicting, incomplete, or suboptimal systems in which patients participate and interfere [43]. The report published by the Institute of Medicine at the beginning of the 21st century resulted in the increased and rapid adoption of health information technology in health care settings, especially for patient safety purposes [44]. For example, the wrong site, wrong side, wrong procedure, and wrong individual (WSWP) errors have been mitigated to some degree by electronic health records. However, these errors continue to be quite significant [45]. DT-assisted safety management systems can be implemented within the Safety 4.0 framework to manage complex safety procedures with minimum human error [46]. In addition to providing operators with contextual information about the surroundings, DTs can guide them through safety tasks [47].

Our study found different DT applications (n=3) that contribute to patient and system safety management through system twinning and product twinning. For instance, an opportunity that DT developers seized was the COVID-19 safety crisis within the health care ecosystem [21]. In 2020, COVID-19 disrupted the health care system and caused an ecosystem crisis that harmed public safety, including resource shortages, misinformation, and medical errors. Virtual interventions and technology initiatives became the preferred mode of service. For example, DTs could accurately contribute to vaccination strategy development. Vaccinations can be targeted based on the number of social contacts of each individual, and infections can be restricted to isolated hotspots and delayed by precisely targeted vaccination, inherent immunity, and public health measures that reduce the infection peak. Thus, DT technology supports decision-making to control the spread of the virus [21]. Apart from this, twinning systems contributed toward predicting the COVID-19 spread. Alrashed et al [23] used a DT system to simulate different strategies and scenarios to minimize the impacts of the virus spread and prevent it while continuing to provide necessary services to citizens with no interruptions to ensure their safety with minimal risk. Safety risk for patients was not only caused by the virus itself but also by the inability of the systems to respond to the new challenges. These challenges will continue to impact the system even after the COVID-19 crisis. Talukder [22] suggested a system architecture that ensures safety within an ecosystem disrupted by COVID-19. It mitigates system-related challenges and increases patient safety in post–COVID-19 health care delivery [22]. In conclusion, DTs (systems and products) can ensure safety management in health care systems by identifying potential threats, redesigning the systems to mitigate hazards, and improving the safety strategies implemented. This leads to the right care at the right price and time for everyone and everywhere at any point of care in a safe manner.

Although the applications of DT technology in patient safety were inspired by COVID-19, it is essential to investigate its potential in other settings and crisis situations. In addition, this technology helps address safety issues in health care without interrupting day-to-day work; it can also be used to address other medical safety issues, such as surgical errors, workplace safety issues, and medical bias in diagnosis.

#### Information Management

The American Health Information Management Association describes health information management as the process of collecting, analyzing, and securing digital and traditional medical records that are vital to providing quality patient care [48]. Health care organizations seek to analyze patients' information efficiently and quickly, both internally and externally [49]. However, they face many challenges such as privacy, exchange restrictions, and extensive data. Increasing amounts of patient data are forcing health care institutions to replace traditional approaches that cannot cope with increases in data. The United States is taking steps to boost health care information and communication access by leveraging advances in information and communication technologies [50]. Health information management systems have grown rapidly in recent years and are being used to derive important health trends and provide timely preventive care [51]. DT technology can revolutionize clinical research with the changes that it can bring to the basis of health care systems and medical practices. By leveraging this technology, users can better ask questions, get better answers, and gain data-driven actionable insights without compromising the health of real-life subjects. In fact, using a DT, people can gather, aggregate, and represent individual information about their health and well-being [52]. As more data are collected, more DTs will be enabled, leading to more discoveries and better treatment, thus allowing the assembly of more data with less cost, and especially eliminating the risks and consent issues associated with actual human subjects [53]. In our review, we found applications related to information management DTs (n=2).

According to Lutze [24], eHealth systems can manage knowledge by implementing DTs that are based on artificial intelligence. He proposed a DT that collects daily activity data from smart assistance systems linked to wearable sensors for elderly people and extracts behavioral knowledge for information management. The technology suggested accounts for systems, processes, and group changes to provide unbiased conclusions based on learned, trained data. It has a human-centered design, as it allows the self-determination and autonomy of patients to share or refuse the usage of their data with providers and clinical staff. It also establishes solid robustness by automatically tracing the use of all knowledge sources and verifying conclusions drawn about patients after system changes [24]. Auditability is established by tracing the use of all knowledge sources and recording and verifying conclusions drawn about patients and users after system changes. Robustness is supported by automatically checking the containment of a patient within the designated user group of the system and verifying the continued validity of the assessed acceptance conditions after system changes. Moreover, human oversight is facilitated in all critical situations [24].

Another example of an information management DT was suggested by Pang et al [25]. During pandemics, sharing information among different cities and countries in real time through a shared learning model (federated learning) remains critical while ensuring enhanced privacy protection. Pang et al used DT technology in a novel collaborative paradigm that allows DTs in multiple cities to share the local strategy and status quickly without violating any privacy rules to help manage the COVID-19 pandemic [25]. These 2 examples show that DTs allow information management and knowledge extraction by encoding, storing, retrieving, and sharing data in a secure, smart, and real-time environment.

#### Health Management and Well-being Promotion

Health and well-being goals are challenging to achieve for many individuals. In response to this challenge, a growing number of technologies are being developed to improve people's diet, physical activity, sleep, and mental health. By promoting behavior change and controlling health care costs through modern digital health interventions, people can maintain better health and a healthier lifestyle. Today, a variety of sensors are miniaturized and widely used to track basic physiological indicators on the move to help with better health and well-being management. Moreover, because smartphones are extremely easy to access, mobile health apps are currently considered the most beneficial platform for promoting healthy lifestyles and changing behavior [54]. In modern medicine, personal health management services are viewed as electronic, remote, and digitally enabled care that helps individuals manage their own care and reduce the need for in-clinic visits that are typically expensive and time-consuming [55].

In our review, we found that DTs were used for health management and well-being promotion (n=3). One such DT was introduced by Díaz et al [26] in 2021. Their DT application was called DTCoach. It is a user-centered smart coach that serves as a mentor and an accompanying educator to the users. It helps the users make the necessary adjustments in their posture and performance based on measurements taken that characterize their individual capabilities [26].

Another example was ClouDTH, suggested by Liu et al [27]. This cloud-based health care system uses personal data from digitally twinned wearable medical devices to achieve a convergent interaction between the medical and physical spaces, and their virtual twins. Then, it facilitates self-management of health for elderly patients [27]. Patients' needs depend on many factors, and age is one of them. Elderly patients have higher demands for many medical services. Therefore, DTs are used extensively in geriatric care to support health management promotion of elderly patients. Furthermore, another example that we cover in our review was introduced by Tröbinger [28], which is a new DT approach serving as an alternative to telemedicine. It consists of a transparent remote doctor visit and a better-informed and robot-assisted telerehabilitation initiative that allows bidirectional telepresence control [28]. In summary, DTs are used to accompany patients and give them control over their health by promoting well-being and lifestyle activities and supervising them to maximize their performance in a safe environment.

#### Operational Control

Operating a health care facility on a day-to-day basis impacts patient experiences and organizational goals [56]. Thus, operations management helps in understanding and optimizing the business processes inside medical departments to reduce and alleviate the effects of overcrowding, waiting times, delays, and other problems that facilities are facing [57]. DTs have the potential to contribute to the effective operation of health care units. Our review found that most DT initiatives to improve health services have health care operations management focusing on revolutionizing clinical processes and enhancing medical care. They replicate hospitals or treatment facilities and help improve their performance in a safe manner with less risk. Applications are numerous and range from predicting resource shortage to managing patient flow. Using DT technology, an institution can execute a digital stress test to observe how the technology would fare under extreme conditions like crises. By creating a virtual twin of a hospital, stakeholders can review the operational strategy, capacity, staffing, and care model on the DT to determine what actions to take and mitigate future challenges. Our review identified extensive efforts (9 out of 17studies) to support operational control and account for health care system challenges. The efforts consisted of digitally twinned systems and processes for performance control.

Detecting anomalies in processes is essential to prevent hazards and predict the corrective actions that need to be implemented. With some interventions, such as the solution suggested by Croatti et al [37], DTs can be used to support physical processes through their digital representations and monitor their changes. In this study, by mirroring the real system by building an agent-based smart DT, they aimed to digitalize and support the process of severe trauma management [37]. This DT represents the operative phase of trauma management and starts when the trauma is marked as severe in the previous phase. The fact that this DT starts before the patient's arrival to the unit is very important for this case study [37]. In this way, the trauma team is prealerted about the incoming patient and starts collecting and receiving information directly from the accident site. Its internal state changes when the patient is delivered to the emergency department, where the trauma team starts taking care of the patient. A very preliminary version of a system prototype has been developed according to the designed conceptual model [37].

Another example is Health@Hand, suggested by Nonnemann et al [29]. They twinned the processes of an intensive care unit (ICU) station and integrated them into a digital system (Health@Hand) to allow remote monitoring of the processes. With this intervention, hospital managers can detect anomalies and faults immediately and intervene in an early stage. Moreover, while improving the productivity and efficiency of processes, some digital interventions forget to address the problem of cybersecurity, which may harm the systems. As a solution to this problem, Mylrea et al [33] propose BioSecure. It is a process twinning that allows managers to monitor every step in the supply chain process to ensure good productivity and cybersecurity to secure the system and data by applying Cyber-Informed Engineering.

One of the challenges in health care is providing an optimized scheduling strategy that can effectively use the hospital's resources and prevent delays, errors, and long lengths of stay. An application in the same settings (ICU and process twins) was developed by Chase et al [30] that aimed to optimize patient care by clinician staff at the enterprise level to improve the productivity of the staff and the quality of care delivered to patients. In addition, using resources effectively has always been a challenging decision for managers in all industries. In health care, resource allocation needs to be regulated by providing efficient services on time. Rodríguez-Aguilar et al [36] suggested a DT for a hospital that supports resource allocation (financial and human) called the Digital Health Public Emergency System (DHPES). DHPES provides efficient health services [36]. The DT design seeks to generate virtual instances that emulate the real operation of the provision of highly specialized public services, including the supply of medications, supplies, devices, and equipment as well as the management of human resources and financial resources in the event of a contingency [36].

Another DT initiative called HospiT'Win was developed to manage the patients' pathways inside the hospitals [34,35]. This DT can help hospitals equilibrate demand and supply and control the growing workload while reducing waiting times, lengths of stay, and delays. It also provides key information to decision-makers in real time, controlling the real-time flow of data. As high demand can disturb health care systems, such DT systems might be useful in times of crisis. Hospit'Win performed well during the COVID-19 pandemic by managing the demand [34,35].

Some operational strategies need to be implemented first to evaluate their efficiency. In hospitals, evaluating interventions would disrupt the daily services and activities of the units. This is where DTs could be most useful. Karakra et al [31] proposed a decision support system that employs real-time services data drawn from real systems and devices to enable evaluation of the impact of changes in services without disrupting the daily activities of the hospital. This idea allows foreseeing the effectiveness of changes in the models before they are applied in reality [31].

Providing patients safe and high-quality care is a demanding process. A DT framework for a system is proposed by Augusto et al [32] to optimize a patient’s care pathway in health care units. The system accounts for various arrival processes and simulates different scenarios to determine the best available leverages to optimize the operations of the system even under high demand and variability to account for uncertainties [32]. The framework has been conceived and tested in close collaboration with health care professionals to be as close to the real system as possible. Furthermore, the framework is intended to be used regularly by the head of the emergency unit [32]. Data collection was performed using the hospital information system for the following parameters: patient arrivals, the total length of stay; type of patient including less critical (fast and normal track), moderately critical, and life-threatening emergencies; and number of requested paraclinical examinations per patient. On the other hand, processing times were recorded by interviewing doctors, nurses, caregivers, and interns because the related data in the hospital information system were not reliable enough [32]. The model was validated and shown to reduce the waiting time and length of stay in different scenarios.

### Future of DTs: Challenges Associated With Use and Implementation

A DT system would provide patients with a safe and secure monitoring option; medical staff would have safe and secure monitoring methods, and authorities would be able to track extreme crisis scenarios in real time accurately. However, digital twinning is facing many challenges that are hindering its growth. The first obstacle is the infrastructure of data flow. For example, to prevent false positives in the digitally twinned sensors, we need a good understanding of the variability in the personal parameters and characteristics of the users [58]. Another concern to be addressed before DTs can go mainstream is data security and privacy. The data used by DTs are confidential and sensitive, and interconnected devices are an easy target for cyberattacks that can harm health care systems. Therefore, governments and policy makers need to consolidate the law regulation factor to have more protected data-sharing procedures. Moreover, as data form the core of DTs, quality control protocols need to be embedded in the real physical systems to merge the data with the simulated systems (twins) and ensure good performance of the DT models. Additionally, ethical concerns like ownership of the data extracted with DTs are still not addressed. Finally, it is essential to explore factors that affect DT implementation and adoption.

DTs also have the potential to offer new important pathways for various care processes in health care. For instance, strategies to improve communication and patient-centered care can be implemented digitally to evaluate their effectiveness before adopting them in real life to avoid repetitive trials that may disturb patients' pathways. In addition, some patients are hard to deal with because of their critical medical situations. Digital twinning of the care processes for these patients can give more visibility to health care professionals to understand the best possible care strategy for these patients. Furthermore, usability studies are sometimes costly in terms of facilities, equipment, and time. DTs can facilitate remote usability testing across diverse populations, accounting for their lower literacy or health literacy and individuals with cognitive or physical disabilities. They can also help testers gain time, reduce effort, and earn money while providing real-time decision support by solving recruitment problems for surveys, interviews, and clinical trials.

**Figure 3:**
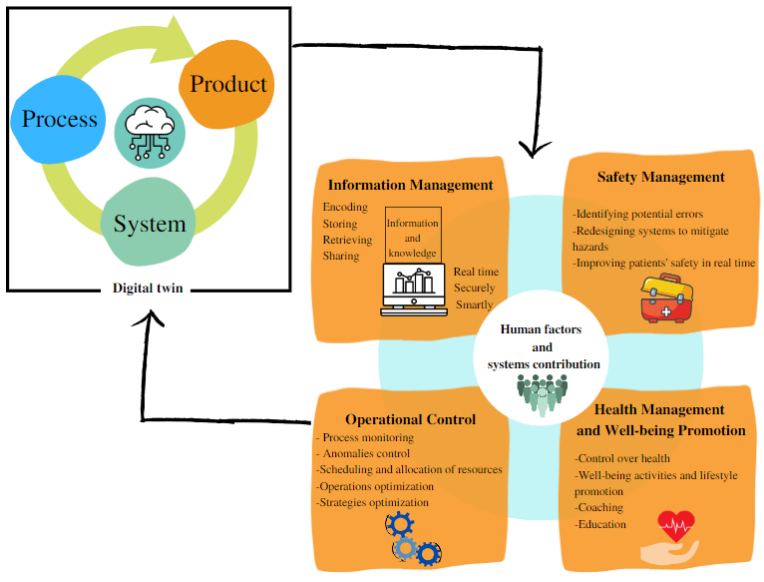
Framework of the impact classification of digital twins from human factors and systems perspectives.

As illustrated in Figure 3, we suggest a framework that highlights the possible contributions of DTs from human factors and systems perspectives. Irrespective of whether the technology designed is by twinning a system, process, or tool, a good DT design can potentially improve safety management, improve operational control of the health care system, allow better information management, and promote the health and well-being of patients.

Finally, this study also has several limitations. The included studies largely reported postintervention data, so we could not determine the preintervention-to-postintervention change or ascertain whether the intervention groups were matched at baseline for key characteristics and outcome measure scores. In addition, we may have missed some articles in our screening because the research was limited to the following databases: PubMed, Web of Science, IEEE Xplore, Scopus, and ScienceDirect.

### Conclusions

DTs are replications of systems, products, or processes that bridge reality using data and expand the same to virtual models. In medical services, DTs are primarily used in personalized medicine; however, they also have the potential to be used at the system level. These applications vary from safety to information management, health and well-being promotion, and operations control. This rapid review shows that digital twinning for health care system management is still an emerging field with considerable potential that was also used during the COVID-19 pandemic. Therefore, interdisciplinary teams from various disciplines, including human factors and ergonomics, human-computer interaction, data science, and digital health, should further investigate the potential of this technology and address the challenges that may influence the design and adoption of this technology in the health care system.

## Abbreviations

DHPES: Digital Health Public Emergency System
DT: digital twin
ICU: intensive care unit
